# Analysis of serum Hsp90 as a potential biomarker of β cell autoimmunity in type 1 diabetes

**DOI:** 10.1371/journal.pone.0208456

**Published:** 2019-01-10

**Authors:** Gail J. Ocaña, Emily K. Sims, Renecia A. Watkins, Susanne Ragg, Kieren J. Mather, Richard A. Oram, Raghavendra G. Mirmira, Linda A. DiMeglio, Janice S. Blum, Carmella Evans-Molina

**Affiliations:** 1 Department of Microbiology and Immunology, Indiana University School of Medicine, Indianapolis, United States of America; 2 Department of Pediatrics and the Herman B Wells Center for Pediatric Research, Indiana University School of Medicine, Indianapolis, United States of America; 3 Department of Obstetrics and Gynecology, Indiana University School of Medicine, Indianapolis, United States of America; 4 Department of Pediatrics, College of Medicine–Jacksonville, University of Florida, Jacksonville, United States of America; 5 Department of Medicine, Indiana University School of Medicine, Indianapolis, United States of America; 6 Institute of Biomedical and Clinical Science and the NIHR Exeter Clinical Research Facility, University of Exeter Medical School, Exeter, United Kingdom; 7 The Roudebush VA Medical Center, Indianapolis, United States of America; Université de Genève, SWITZERLAND

## Abstract

Heat shock protein 90 (Hsp90) is a protein chaperone that is upregulated and released from pancreatic β cells under pro-inflammatory conditions. We hypothesized that serum Hsp90 may have utility as a biomarker of type 1 diabetes risk and exhibit elevations before the onset of clinically significant hyperglycemia. To this end, total levels of the alpha cytoplasmic isoform of Hsp90 were assayed in autoantibody-positive progressors to type 1 diabetes using banked serum samples from the TrialNet Pathway to Prevention Cohort that had been collected 12 months prior to diabetes onset, with comparison to age, sex, and BMI-category matched autoantibody-positive nonprogressors and healthy controls. Hsp90 levels were higher in autoantibody-positive progressors and nonprogressors ≤ 18 years of age compared to matched healthy controls. However, Hsp90 levels were not different between progressors and nonprogressors in any age group. Hsp90 was positively correlated with age in control subjects, but this correlation was absent in autoantibody positive individuals. In aggregate these data indicate that elevated Hsp90 levels are present in youth with β cell autoimmunity, but are not able to distinguish youth or adult type 1 diabetes progressors from nonprogressors in samples collected 12 months prior to diabetes development.

## Introduction

Type 1 diabetes (T1D) has been defined classically as a T cell-mediated autoimmune disease in which insulin-producing β cells are targeted for destruction by the host immune system. At present, a lack of reliable biomarkers remains a major obstacle in both the identification of individuals at-risk of developing T1D and in the implementation of disease modifying therapies [1]. Recent data have highlighted a role for β cell stress pathways such as endoplasmic reticulum stress and oxidative stress that may synergize with the immune system to accelerate T1D progression [2, 3]. Activation of these pathways is thought to precede the development of clinically detectable hyperglycemia [4], thus raising the possibility that biomarkers of β cell health may have utility in disease prediction and treatment paradigms.

Heat shock protein 90 (Hsp90) is a highly-conserved member of the heat-shock family of molecular chaperone proteins. Intracellularly, these chaperones assist a wide variety of protein clients in the acquisition of active conformations using energy derived from ATP binding and hydrolysis [5]. Islet Hsp90 levels were shown recently to be elevated in NOD mice prior to the onset of hyperglycemia [6]. In other cell types, intracellular Hsp90 expression and Hsp90 release are increased in response to reactive oxygen species and other environmental stressors such as hypoxia and irradiation [7]. Consistent with these findings, cadaveric human islets and pancreatic β cell lines released increased levels of Hsp90 in response to pro-inflammatory cytokine treatment [8, 9]. Moreover, pediatric subjects with recent-onset T1D demonstrated elevated serum levels of the alpha cytoplasmic isoform of Hsp90 compared to age and sex-matched controls [6], while elevated levels of circulating IgG1 and IgG3 class-switched anti-Hsp90 autoantibodies have been identified in individuals with T1D [10].

Taken together, these data suggest that extracellular and potentially β cell-derived Hsp90 may be indicative of ongoing β cell stress and islet inflammation during the evolution of T1D. We therefore hypothesized elevations in serum Hsp90 levels may be present prior to the onset of clinical symptoms of diabetes and serve to predict T1D development. To this end, serum Hsp90 levels were assayed in samples from the TrialNet Pathway to Prevention (PTP) cohort, which is a longitudinal study of at-risk, autoantibody-positive relatives of individuals with T1D who are prospectively monitored for the development of dysglycemia and diabetes. Serum Hsp90 was measured in individuals who progressed to T1D in samples collected approximately 12 months prior to the onset of clinical disease, and levels were compared to age-, sex-, and BMI-matched healthy controls and autoantibody positive nonprogressors who remained diabetes-free.

## Methods

### Biobanked Serum Samples

TrialNet is an ongoing clinical trial with centers in the United States, Canada, United Kingdom, Germany, Italy, Australia, and New Zealand. The TrialNet Pathway to Prevention (PTP) study (TN01; clinical trial reg. no. NCT00097292, clinicaltrials.gov) longitudinally monitors autoantibody positive first-, second-, and third-degree blood relatives of persons with T1D for changes in autoantibody status, dysglycemia, and progression to T1D [11]. Serum is regularly collected and banked at the time of oral glucose tolerance testing. For this study, we analyzed stored serum samples collected from 60 autoantibody-positive TrialNet PTP study participants collected approximately 12 months before the development of type 1 diabetes. Diabetes was diagnosed according to criteria established by the American Diabetes Association [12]. To approximate prepubertal, peripubertal, and postpubertal age groups, equal numbers of progressors were chosen from three age ranges (< 10, 10–18, and > 18 years of age). In addition, banked serum samples were obtained from autoantibody-positive PTP participants who did not progress to T1D with the same type and length of monitoring. These autoantibody-positive nonprogressors were chosen to match progressors based on age, sex, and BMI/BMI z scores. Banked pediatric serum samples from age, gender, and BMI z-score healthy controls were obtained locally at Indiana University from ambulatory dental and eye clinics. Control pediatric subjects did not take any chronic prescription medications, had no chronic health diagnoses or family history of diabetes, and had no acute febrile illness within two weeks preceding sampling. Banked adult serum samples were also obtained at Indiana University from age, gender, and BMI-matched healthy controls who were recruited on the basis of a normal response during an oral glucose tolerance test. Exclusion criteria for adult controls included a history of any type of diabetes, current pregnancy, weight fluctuation in the preceding 6 months, current or past tobacco use, acute or chronic illness, pulmonary disease, or use of antidepressants, metformin, or thiazolidinediones.

TrialNet protocols were approved by local Institutional Review Boards. Collections at Indiana University were approved by the Indiana University Institutional Review Board. Written informed consent or parental consent and child assent were obtained from all participants before any research participation in accordance with the ethical guidelines of each institution. TrialNet consents explicitly allowed use of stored samples in ancillary studies such as the current project.

### Hsp90 ELISA and T1D Autoantibody Assays

Total serum levels of the alpha cytoplasmic isoform of Hsp90 were measured by ELISA (Enzo Life Sciences). This assay detected Hsp90 levels in the range of 62.5–4000 pg/mL, with a reported sensitivity of 50 pg/mL and no cross-reactivity with the beta cytoplasmic isoform of Hsp90, Grp94, Hsp60, or Hsp70. Autoantibodies to GAD65 or GAD65H, microinsulin antibodies (mIAAs), islet cell antibodies (ICAs), or IA-2H were measured according to methods outlined by the Diabetes Antibody Standardization Program as described previously [11]. Given that TrialNet recently replaced GAD65 and ICA512 with GAD65H and IA-2H assays, individuals with positive results for either assay were considered positive for GAD or ICA autoantibodies.

### Statistics

Because several sets of data in our analysis were not normally distributed according to D’Agostino-Pearson omnibus normality tests, continuous data were graphed as medians with interquartile ranges (IQRs) and analyzed using non-parametric statistical tests. Non-parametric tests are less sensitive to outlier points and are more suitable for data derived from non-normal distributions [13]. For comparisons between two groups, statistical significance was determined using a two-tailed Mann-Whitney test. To determine statistical significance among three groups, the Kruskal-Wallis test was used with Dunn’s test to correct for multiple comparisons. Receiver operating characteristic (ROC) analysis was performed to determine area under the curve (AUC) using Stata 14 (Statacorp LP, Texas, USA). Spearman correlations were used to analyze relationships between serum Hsp90 levels and demographic variables. Categorical variables were compared among groups by Pearson’s chi-square tests. *P*-values of < 0.05 were considered statistically significant. Statistics were calculated using GraphPad Prism 7.0c (GraphPad Software). The complete dataset is included in the Supporting Information.

## Results

Characteristics for the 60 control subjects, 60 autoantibody-positive TrialNet nonprogressors, and 60 autoantibody-positive TrialNet progressors are shown in Table 1. No differences in age, sex, or BMI *z*-scores were observed between the groups. Within the progressor and nonprogressor PTP groups, 56 subjects tested positive for 0–1 autoantibodies, while 64 subjects tested positive for two or more autoantibodies. As expected, a higher percentage of progressors were positive for two or more autoantibodies compared to the nonprogressors (*P* < 0.0001).

**Table 1:** Baseline Characteristics of Study Participants.

Variable: median (IQR)	Aab Negative (n = 60)	Non-Progressors (n = 60)	Progressors (n = 60)	*P* value
Age (years)	12.0 (8.0, 25.8)	12.0 (7.3, 25.0)	11.6 (7.8, 26.5)	*ns*
Male sex (%)	48.3	48.3	48.3	*ns*
BMI—for—age (z-score)	0.76 (-0.12, 1.73)	0.76 (-0.01, 1.28)	0.68 (-0.13, 1.42)	*ns*
Multiple Aab positive (%)	0	40.0	66.7	< 0.0001

Median serum Hsp90 values by age category for autoantibody-negative control subjects were as follows: 11.8 ng/mL (IQR 8.2, 15.4) in subjects < 10 years of age, 8.4 ng/mL (IQR 7.3, 11.1) in subjects 10–18 years of age, and 17.9 ng/mL (IQR 11.1, 22.7) in subjects > 18 years of age. In the autoantibody positive non-progressor group, median serum Hsp90 values were 21.3 ng/mL (IQR 15.2, 27.4) in subjects < 10 years of age, 20.9 ng/mL (IQR 13.6, 26.1) in subjects 10–18 years of age, and 19.6 ng/mL (IQR 14.9, 28.1) in subjects > 18 years of age. For progressors, median serum Hsp90 values were 22.0 ng/mL (IQR 17.2, 33.6) in subjects < 10 years of age, 19.4 ng/mL (IQR 10.5, 26.2) in subjects 10–18 years of age, and 16.5 ng/mL (IQR 10.2, 24.3) in subjects > 18 years of age.

**Fig 1:**
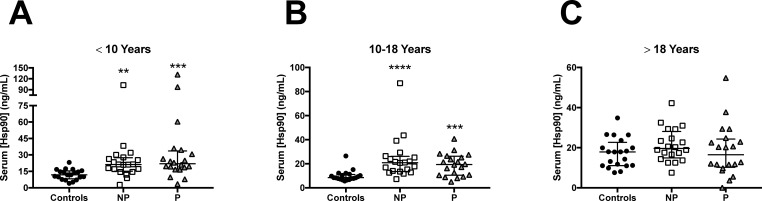
Serum Hsp90 values in TrialNet PTP study participants. Scatterplots of serum Hsp90 values for subjects < 10 years of age; n = 20 in each group (A), 10–18 years of age; n = 20 in each group (B), and > 18 years of age; n = 20 in each group (C). Kruskal-Wallis tests demonstrated significant differences between groups for subjects < 10 years of age (*P* = 0.0001) and 10–18 years of age (*P* < 0.0001), but not for subjects > 18 years of age. Dunn’s multiple comparisons tests revealed significant differences between autoantibody-positive nonprogressors and progressors compared to autoantibody-negative controls in subjects < 10 years of age (*P* = 0.0017 and *P* = 0.0004, respectively) and 10–18 years of age (*P* < 0.0001, *P* = 0.0001, respectively). No differences were observed between nonprogressor and progressor subjects for either age group. **P < 0.01, ***P < 0.001, ****P < 0.0001. NP: nonprogressor, P: progressor.

Comparison between groups showed that serum Hsp90 levels were significantly higher in autoantibody-positive nonprogressors and progressors compared to control subjects in persons < 10 years of age (*P* = 0.0017 and *P* = 0.0004, respectively) and 10–18 years of age (*P* < 0.0001, *P* = 0.0001, respectively). In these age groups, however, no differences in HSP90 levels were detected between autoantibody-positive nonprogressor and progressor subjects (Fig 1A and 1B). Analysis of subjects > 18 years of age revealed no differences in Hsp90 levels among the three groups (*P* = 0.273) (Fig 1C).

**Fig 2:**
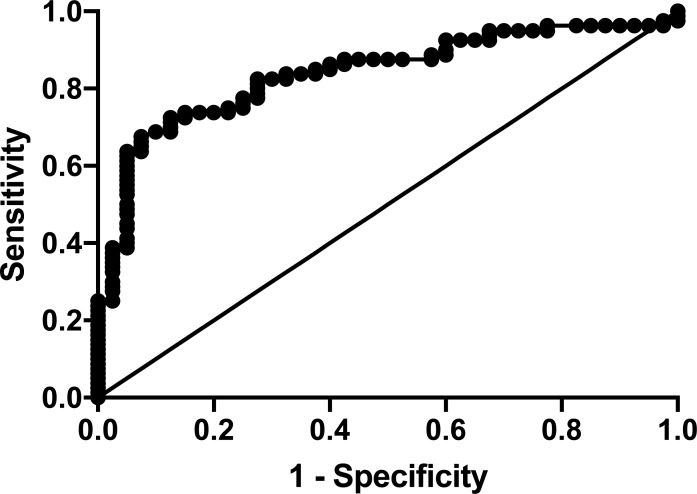
ROC curve analysis of serum Hsp90 levels to predict autoantibody-positivity in subjects ≤ 18 years of age. ROC analysis was performed to determine the utility of serum Hsp90 levels as a predictor of autoantibody-positivity in subjects ≤ 18 years of age. (AUC: 0.84; 95% CI: 0.77–0.91; *P* < 0.0001).

ROC analysis was used to examine the discriminative ability of serum Hsp90 levels in distinguishing controls from autoantibody-positive subjects ≤ 18 years of age. An area under the curve (AUC) of 1 in ROC analysis represents perfect discrimination, while an AUC of >0.8 is considered a clinically useful test [14]. The area under to curve (AUC) for Hsp90 was calculated to be 0.84 [95% confidence interval (CI): 0.77–0.91; *P* < 0.0001] (Fig 2), confirming the ability of Hsp90 to distinguish youth with autoimmunity from a healthy control population.

**Fig 3:**
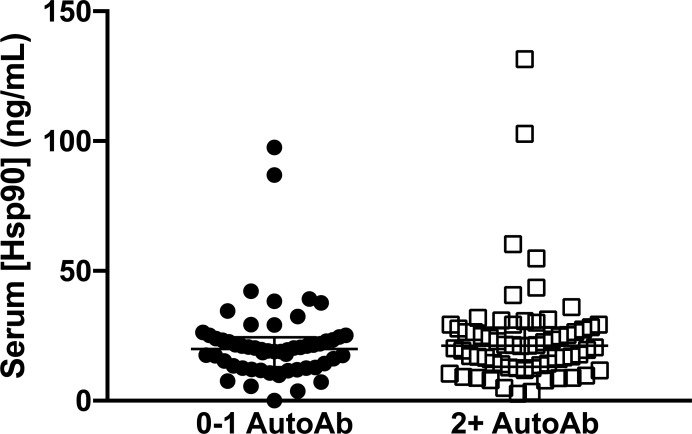
Relationship of serum Hsp90 levels with islet autoimmunity. Scatterplot comparing serum Hsp90 levels in subjects testing positive for 0–1 (*n* = 56) and two or more (*n* = 64) islet autoantibodies (AutoAb). No difference was observed by Mann-Whitney test (*P* = 0.57).

To determine whether serum Hsp90 levels were different in those with greater numbers of detectable islet autoantibodies, serum Hsp90 levels were assessed relative to autoantibody status. No differences in serum Hsp90 levels were detected between progressor and nonprogressor subjects positive for 0–1 autoantibody compared to those who were positive for two or more autoantibodies (*P* = 0.57) (Fig 3).

**Fig 4:**
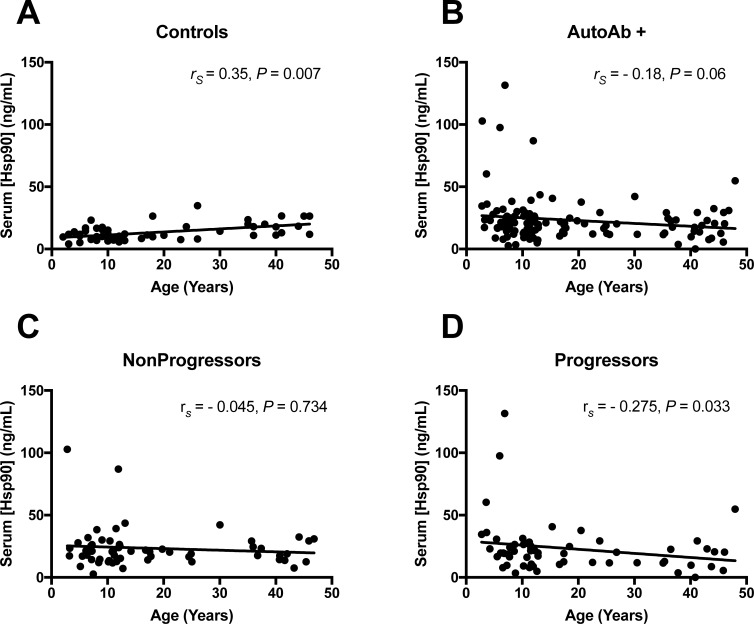
Correlations of serum Hsp90 levels with age. Spearman correlation coefficients were generated to test correlation of serum Hsp90 levels with age in *n* = 60 autoantibody-negative (A) and *n* = 120 autoantibody-positive (B) subjects and then separately for the nonprogressors (n = 60) (C) and progressors (n = 60) (D). A significant positive correlation between serum Hsp90 levels and age was observed in autoantibody-negative subjects (*r*_*s*_ = 0.35, *P* = 0.007), while serum Hsp90 levels exhibited a weak trend toward decreasing with age in autoantibody-positive subjects (*r*_*s*_ = -0.18, *P* = 0.06). No significant relationship with age was observed in the nonprogressor group (*r*_*s*_ = -0.045, *P* = 0.734). However, there was a significant negative inverse correlation with age in the progressors to type 1 diabetes (*r*_*s*_ = -0.275, *P* = 0.033).

Because we found that Hsp90 levels were elevated in pre-pubertal and pubertal subjects (Fig 1), we also examined correlations between serum Hsp90 levels and age. In control subjects, serum Hsp90 levels were significantly higher with age (*r*_*s*_ = 0.35, *P* = 0.007) (Fig 4A). Conversely, serum Hsp90 levels exhibited a weak inverse correlation with age in autoantibody-positive subjects (*n* = 120) (*r*_*s*_ = -0.18, *P* = 0.06) (Fig 4B). We analyzed correlations with age separately for the nonprogressors (Fig 4C) and progressors (Fig 4D). There was a significant negative inverse correlation with age in the progressors to type 1 diabetes (*r*_*s*_ = -0.275, *P* = 0.033), whereas no significant relationship between serum Hsp90 levels and age was observed in the nonprogressors (*r*_*s*_ = -0.045, *P* = 0.734). No significant correlations were found between serum Hsp90 levels and sex or BMI/BMI *z*-scores (data not shown).

## Discussion

Hsp90 is an attractive biomarker of T1D risk based on a growing body of evidence implicating islet β cell endoplasmic reticulum (ER) stress in T1D pathogenesis. Protein markers of ER stress are upregulated in islets of humans with T1D [15]. In addition, ER stress is known to precede the development of T1D in NOD mice [16], while mitigation of ER stress with chemical chaperones prevented the development of T1D in NOD mice [17]. Pro-inflammatory cytokines are released locally within the pancreas by activated macrophages, natural killer cells, and T cells during the course of insulitis [9]. Ex vivo treatment of human cadaveric islet cells and β cell lines with pro-inflammatory cytokines has been shown also to induce ER stress [18]. Consistent with this, we previously found that pro-inflammatory cytokines induced release of Hsp90 from β cells [8] and that HSP90 protein expression is elevated in islets from pre-diabetic NOD mice at a time when ER stress is known to be present. Moreover, we have shown that serum Hsp90 levels were elevated in pediatric subjects at the time of T1D diagnosis [6]. Whether Hsp90 levels are elevated prior to a clinical diagnosis of Stage 3 T1D, therefore serving as a biomarker of T1D risk, has not been tested.

To this end, we measured serum levels of Hsp90 in samples obtained from T1D progressors and nonprogressors followed in the TrialNet Pathway to Prevention Cohort. We tested a timepoint 12 months before diabetes onset, because natural history studies have identified this as a period of accelerated loss of C-peptide and rising glycemia [19–21]. We found that serum Hsp90 levels were higher in autoantibody-positive subjects < 10 years of age and 10–18 years of age compared to healthy control subjects (Fig 1A and 1B). However, Hsp90 levels were not different between autoantibody positive T1D progressors and nonprogressors who were followed for an equivalent length of time (Fig 1A–1C). In addition, levels of Hsp90 were not increased in progressors or nonprogressors over age 18 compared to matched controls (Fig 1C).

Hsp90 is expressed in a number of tissues, including immune cells. Indeed, studies have demonstrated elevations in serum Hsp90 levels in a variety of other autoimmune and inflammatory conditions. In a cohort of individuals with bullous pemphigoid, skin as well as peripheral blood mononuclear cell (PBMC) levels of Hsp90 were elevated relative to controls [22]. PBMC Hsp90 levels were also elevated in systemic lupus erythematosus, and levels correlated with increased levels of circulating IgG autoantibodies to Hsp90 [23]. Thus, elevations in serum Hsp90 levels may reflect more generalized states of inflammation as might be expected in individuals with β cell autoimmunity.

Analyses were performed to explore whether elevations in Hsp90 were related to key demographic and clinical variables. We did not detect a difference in Hsp90 levels by number of autoantibodies in the progressor and nonprogressor groups (Fig 3). This could indicate that Hsp90 becomes elevated early during autoimmunity and remains chronically elevated without significant fluctuation with the intensity of autoimmunity. Future longitudinal analysis would be needed to fully test this possibility. Interestingly, Hsp90 levels exhibited a decreasing trend with age in autoantibody-positive subjects and a significant decreasing trend with age in the progressors to type 1 diabetes (Fig 4B and 4D). These results were similar to our previous study examining correlations between age and serum Hsp90 level in children aged 4–15 years with new-onset T1D [6] and could be indicative of the aggressive β cell autoimmunity typically experienced by younger individuals [24, 25]. Related to this, levels of HSP90 were not different in older progressors >18 years compared to matched controls (Fig 1C). Notably, we observed increasing serum Hsp90 levels with age in control subjects (Fig 4A), and these age-related elevations may have obscured any differences in serum Hsp90 levels that were associated with autoantibody status in this older age group. Associations between aging and inflammation are well-established, and increased expression of inflammatory markers has been referred to as “inflammaging”, a process thought to contribute to accelerated aging and decreased lifespan in older adults [26]. Interestingly, Hsp90 inhibitors were shown recently to have therapeutic efficacy to extend lifespan, acting as a senolytic agent in rodents [27]. Therefore, the possibility remains that elevated baseline levels of serum Hsp90 in older individuals reveal the increased inflammation associated with aging, while the absence of these age-related elevations make Hsp90 a more suitable biomarker to detect pathological inflammatory processes in younger individuals.

In summary, this study was the first to examine whether elevations in serum Hsp90 levels precede T1D development. Our results indicate that elevated serum levels of Hsp90 may be associated with β cell autoimmunity in pediatric patients. However, differences in Hsp90 levels were not able to predict whether or not an autoantibody-positive individual will develop T1D within a 12-month timeframe.

## Supporting information

S1 File Complete dataset. Included in the supporting information is an excel file with the complete dataset used for analysis.
(XLSX)
